# The Cognitive Cost of Motor Control: A Systematic Review and Meta-Analysis of Parkinson’s Disease Treatments and Financial Decision-Making

**DOI:** 10.3390/healthcare13151850

**Published:** 2025-07-29

**Authors:** Nektaria Kandylaki, Panayiotis Patrikelis, Spiros Konitsiotis, Lambros Messinis, Vasiliki Folia

**Affiliations:** 1 Laboratory of Neuropsychology and Behavioral Neuroscience, School of Psychology, Aristotle University of Thessaloniki, University Campus, 54124 Thessaloniki, Greecelmessinis@psy.auth.gr (L.M.); 2 Department of Neurology, Faculty of Medicine, School of Health Sciences, University of Ioannina, 45110 Ioannina, Greece

**Keywords:** Parkinson’s disease, financial capacity, treatment, levodopa, dopamine agonists, deep brain stimulation, systematic review, meta-analysis

## Abstract

**Background:** Despite growing interest in the literature on Parkinson’s disease (PD) on cognitive functioning, financial incompetence—a crucial aspect of daily living—and its modulation susceptibility by PD treatment regimens remains relatively understudied. **Objective:** This systematic review and meta-analysis aimed to synthesize existing evidence on how PD treatments affect financial capacity, assessing both direct financial competence and cognitive or behavioral proxies of financial decision-making. **Methods:** A comprehensive literature search according to PRISMA protocol was conducted across major biomedical databases, supplemented by gray literature and manual reference list checks. Eligible studies assessed financial capacity directly or indirectly through cognitive proxies (e.g., executive function, decision-making) or financial risk behaviors (e.g., impulse control disorders). Two separate meta-analyses were performed. Heterogeneity (I^2^), publication bias (Egger’s test), and sensitivity analyses were conducted to assess robustness. **Results:** Twenty-three studies met inclusion criteria. One study directly measured financial capacity and was analyzed narratively, reporting diminished competence in patients on levodopa therapy. A meta-analysis of cognitive proxies (10 studies) showed a moderate effect size (Hedges’ g = 0.70, 95% CI [0.45, 0.92], *p* < 0.001), indicating that PD treatments negatively affect executive function and financial decision-making. A second meta-analysis of impulse control and financial risk behaviors (12 studies) revealed a larger effect size (Hedges’ g = 0.98, 95% CI [0.75, 1.22], *p* < 0.001), strongly linking dopamine agonists to increased financial risk-taking. Moderate heterogeneity (I^2^ = 45.8–60.5%) and potential publication bias (Egger’s test *p* = 0.027) were noted. **Conclusions:** These findings suggest that PD treatments negatively impact financial decision-making both directly and indirectly through cognitive and behavioral pathways. Integrating financial decision-making assessments into PD care, particularly for patients on dopamine agonists, is recommended. Future research should prioritize longitudinal studies and standardized neuropsychological measures to guide clinical practice and optimize patient outcomes.

## 1. Introduction

Parkinson’s disease (PD), along with other chronic conditions, is among the most commonly diagnosed and extensively studied neurological disorders. It is estimated that millions of individuals are affected by PD, and the number of cases is expected to rise gradually with increasing life expectancy [1]. PD is a progressive neurodegenerative disorder characterized by the loss of dopaminergic neurons in the substantia nigra of the basal ganglia, impairing the motor circuits of the basal ganglia–thalamocortical pathway. Neuronal degeneration disrupts dopamine signaling, leading to a range of motor and non-motor symptoms that significantly impact quality of life.

Regarding motor control, as dopamine depletion progresses, compensatory mechanisms fail, which results in decreased motor control. Bradykinesia, rigidity, resting tremor, and postural instability are hallmark motor symptoms of PD, all of which contribute substantially to disability [2]. These motor symptoms frequently dominate the early clinical presentation and serve as key diagnostic criteria [3].

However, non-motor symptoms typically emerge earlier in the course of PD [4] and may develop up to a decade before motor symptoms [5]. Non-motor symptoms, including cognitive decline, mood disorders, and sleep disturbances, further exacerbate disease burden and often prove more disabling than motor symptoms, particularly in advanced stages, thereby complicating disease management [6]. Common non-motor manifestations include neuropsychiatric disturbances, gastrointestinal dysfunction, autonomic impairments, and cognitive deficits, with the latter being particularly detrimental [3,7].

Cognitive impairments in PD frequently affect decision-making, working memory, and risk assessment, functions essential for everyday activities, including financial decision-making. When compromised, these cognitive domains can lead to poor financial judgment and increased vulnerability to exploitation [8]. The pathophysiology of PD, therefore, can impair patients’ ability to manage their financial affairs—a critical component of personal autonomy—and adversely affect their overall quality of life [9,10,11]. This cognitive decline has multiple etiologies, stemming not only from disruptions in dopaminergic pathways, but also from impairments in other neurotransmitter systems, such as cholinergic and serotonergic circuits [6]. These deficits are further exacerbated by disease progression and the side effects of pharmacological treatments [12].

Treatment for Parkinson’s disease (PD) primarily aims to alleviate motor symptoms, as no cure currently exists. Managing PD involves developing complex medication regimens tailored to individual patients’ needs [13], which are often expensive and challenging to maintain [14]. Levodopa, dopamine agonists (e.g., pramipexole, ropinirole), and MAO-B inhibitors remain the cornerstone of treatment. As the disease progresses, patients typically require more advanced therapies such as continuous subcutaneous apomorphine infusion, levodopa–carbidopa intestinal gel infusion, or deep brain stimulation. These therapies are relatively costly and can strain financial resources [15]. Although treatment can significantly impact patients’ financial capacity, this aspect has received relatively little clinical or ethical attention in the dementia literature [16]. The choice of treatment for early-stage Parkinson’s disease must therefore be carefully weighed against financial considerations, among other factors [17]. Consequently, treatment decisions can directly influence a patient’s financial situation.

In addressing motor symptoms, levodopa—typically administered with carbidopa—is considered the treatment of choice. However, prolonged levodopa treatment may lead to motor fluctuations and dyskinesia, and its impact on cognition varies. Some individuals experience improvements in attention and working memory with levodopa, while others, particularly as the disease progresses, experience cognitive fluctuations due to the “on-off” phenomenon [18,19].

Dopamine agonists such as pramipexole and ropinirole, though effective in managing early PD symptoms, present substantial drawbacks, including the risk of impulse control disorders (ICDs). ICDs may manifest as pathological gambling, excessive spending, and other risky financial behaviors, reflecting dysfunctions in the mesolimbic dopamine system and compromising financial decision-making [20,21,22]. Patients may overvalue immediate rewards over larger, delayed ones [23,24]. Even in the absence of clinically apparent ICDs, individuals with PD tend to make more impulsive financial decisions regardless of medication status, suggesting either a persistent effect of dopaminergic therapy or an intrinsic change in decision-making behavior caused by PD itself [23].

Deep brain stimulation (DBS) is another widely employed intervention, particularly for advanced-stage PD. By electrically modulating structures such as the subthalamic nucleus, DBS targets motor symptoms, providing significant relief and improving quality of life. However, its effects on cognitive abilities are unpredictable. While some patients experience stabilization or delay in cognitive decline, others face worsening executive function, potentially impairing their financial decision-making abilities [25,26].

Non-pharmacological interventions offer additional pathways to preserve executive function and attenuate the decline in financial capacity. Cognitive training and behavioral therapies, along with physical exercise—which is thought to promote neuroplasticity—have been shown to significantly benefit cognitive resilience in PD patients [27]. The COVID-19 pandemic further highlighted the importance of physical therapy interventions in PD management, as the absence of these services during lockdowns led to neurological deterioration, which may have indirectly impacted financial capacity by undermining patients’ ability to work or manage daily tasks [28].

Ultimately, the effects of various treatments on financial capacity, combined with their cognitive impacts, demand a more comprehensive understanding of how therapeutic decisions influence the overall lives of patients with PD.

Financial capacity refers to an individual’s ability to independently manage financial resources and tasks, involving skills such as budgeting, bill payment, investment decision-making, and fraud detection [8]. In the context of Parkinson’s disease (PD), financial capacity is often compromised by cognitive and motor impairments, as well as the psychological effects of the disease [8]. Cognitive impairments arise from deficits in decision-making abilities and executive functions, which are linked to dysfunctions in the prefrontal cortex, anterior cingulate cortex, and basal ganglia. For example, reduced dopamine signaling in the dorsolateral prefrontal cortex impairs higher-order cognitive functions such as planning and risk assessment. Similarly, dysfunction in the orbitofrontal cortex and ventromedial prefrontal cortex disrupts reward processing and decision-making, while hippocampal deficits impair memory retention, further undermining financial skills. Collectively, these deficits lead to errors in financial transactions, increased vulnerability to financial exploitation, risky and impulsive behavior, and dependence on caregivers for financial management [8,10]. In this review, we distinguish three related but conceptually distinct domains: *financial capacity* (real-world monetary task management), *financial decision-making* (cognitive evaluation of monetary options), and *financial risk-taking* (impulsive or reward-driven monetary behaviors, often observed in the context of impulse control disorders) (see Table 1 for conceptual distinctions). While these domains are functionally interconnected and may share underlying neurocognitive mechanisms, they engage different psychological processes and have different clinical implications. Notably, many studies in the existing literature—reflecting broader trends in neuropsychology and behavioral neurology—use these terms interchangeably, often without clearly delineating their scope or measurement focus. This terminological ambiguity complicates direct comparison and synthesis across studies. In this review, we aim to clarify these distinctions where possible and categorize the findings accordingly to support conceptual precision and interpretative clarity.

As cognitive capacity declines in PD, impairments in financial capacity progress in parallel. Initially, patients may struggle with complex financial tasks such as investing or long-term planning. Over time, these difficulties extend to more basic financial activities, including bill payment and fraud detection. In the later stages, dementia or severe cognitive impairment typically necessitates complete reliance on caregivers for financial management [8].

Previous research also indicates that the side effects of PD treatments can further impair financial decision-making. Although dopaminergic treatments alleviate motor symptoms, they have also been associated with increased impulsivity and risk-taking behaviors, such as gambling addiction and compulsive shopping [29,30,31]. These manifestations suggest a compromise in patients’ decision-making capabilities, underscoring the need for comprehensive research to evaluate how various treatments impact financial capacity.

Considering the strong influence of PD on many aspects of functioning—including financial capacity—and the complexity of managing motor symptoms, it is crucial to understand the extent to which various treatment modalities can mitigate these impairments and affect patients’ ability to maintain financial independence. Previous research in the field of Parkinson’s disease has explored cognitive and financial aspects independently [8,21], but few attempts have been made to provide a cohesive assessment of their connection to treatment regimens [32]. Consequently, the direct effects of available interventions on financial capacity remain under-addressed [33,34]. This meta-analysis seeks to fill this gap by systematically analyzing and synthesizing evidence of the effects of various Parkinson’s Disease treatments and their impacts on financial capacity in adults with PD, compared to PD patients on different or no such treatments and/or to healthy controls, with the ultimate goal of guiding clinical decision-making and optimizing patient care outcomes.

## 2. Materials and Methods

This narrative review and meta-analysis examined the impact of PD treatment on financial capacity. Financial capacity, defined as the ability to manage monetary transactions, make sound financial decisions, and engage in long-term financial planning, is a multifaceted construct influenced by cognitive, executive, and impulse control processes. Given the literature-supported impact that dopaminergic treatment and deep brain stimulation (DBS) can have on cognitive function and risk-taking behaviors, thoroughly investigating their effects on financial decision-making is critically important.

This review adhered to the PICO framework to ensure a systematic and structured approach for study selection and data synthesis. Although the original aim was to conduct a comprehensive meta-analysis, the limited availability of studies directly measuring financial capacity in patients with PD necessitated an integrated approach. Since only one study directly assessed financial capacity using a validated instrument, a narrative synthesis was conducted. Subsequently, two separate groups were formulated: (1) Cognitive Proxies of Financial Capacity (decision-making, executive function, and risk-taking) and (2) impulse control and risky financial-related behaviors. Two different meta-analyses were performed to infer impairments in financial decision-making and behavioral risks contributing to financial mismanagement, respectively.

All meta-analytic computations—including pooled effect sizes, heterogeneity estimates (Cochran’s Q, I^2^), sensitivity analyses, and publication bias tests (Egger’s regression, funnel plots)—were performed using R version 4.2.2 with the metaphor and meta packages. Forest plots, funnel plots, and leave-one-out sensitivity analyses were generated using these tools under a random-effects model framework. These tools are free and open source, ensuring full replicability of the statistical procedures employed.

Although no formal protocol was preregistered or published prior to conducting this review, it was conducted retrospectively and strictly adhered to PRISMA guidelines to ensure methodological rigor in study selection, data extraction, and statistical analyses. Recognizing the importance of preregistration in enhancing transparency and reproducibility, we are committed to registering protocols for future systematic reviews in accordance with best practices for transparency and reproducibility.

### 2.1. Search Strategy

To identify studies examining the effects of treatment on the financial capacity of individuals with Parkinson’s disease (PD), a comprehensive, multi-database literature search was conducted. The search strategy and inclusion criteria were determined before the data analysis. The search was conducted manually using the native interfaces of each database (PubMed, Cochrane Library, Embase via ScienceDirect, Web of Science, ERIC via EBSCO, eBook Collection via EBSCOhost, Scopus, and ProQuest) and was completed in November 2024. No automation tools or version-specific software were used. To minimize publication bias, supplementary searches were performed using gray literature sources, including Google Scholar (screening the first 10 pages of results), conference proceedings, ResearchRabbit, and manual searches of reference lists from the included studies.

The search strategy incorporated Boolean operators (AND, OR), truncation (e.g., “financ*”), and database-specific indexing terms (e.g., MeSH in PubMed) to optimize retrieval. Search terms were iteratively refined to maximize sensitivity while maintaining specificity. To maintain linguistic consistency and ensure accurate interpretation, the review was restricted to journals published in English and Greek. The complete database-specific search strings are provided in Supplementary Material S1.

### 2.2. Eligibility Criteria

Studies were deemed eligible if they examined adults aged 18 years or older diagnosed with PD and assessed the effects of anti-parkinsonian interventions (pharmacological treatments, such as dopamine agonists or levodopa, or non-pharmacological treatments targeting PD symptoms, such as deep brain stimulation). Comparisons included de novo PD patients not yet receiving treatment, individuals with PD receiving alternative therapies, or within-subject pre–post comparisons evaluating treatment effects over time.

Eligible studies were required to measure either financial capacity explicitly (e.g., using validated tools such as the Financial Capacity Instrument or LCPLTAS) or to assess related constructs indirectly. Indirect assessments were accepted if they evaluated financial decision-making (e.g., cognitive tasks involving reward evaluation and probabilistic reasoning) or financial risk-taking behaviors (e.g., pathological gambling or impulsivity-related monetary behaviors). These constructs, while related, were classified distinctly to preserve conceptual clarity.

Only studies employing a quantitative research design were considered. Eligible methodologies included randomized controlled trials (RCTs), cohort studies, and case-control studies. Studies that did not incorporate any measures of financial capacity, whether direct or indirect, were excluded. Qualitative studies, systematic reviews, meta-analyses, and theoretical papers were also excluded. Single-case reports were included only if they provided a quantifiable financial capacity outcome assessed using a validated instrument; purely descriptive case reports were not considered.

### 2.3. Study Selection

The study selection process followed the Preferred Reporting Items for Systematic Reviews and Meta-Analyses (PRISMA) guidelines [35] to ensure methodological transparency and reproducibility. Two independent reviewers screened the titles and abstracts of all identified records to assess their relevance based on predefined eligibility criteria. Any study that could not be definitively excluded at the abstract level was subjected to a full-text review.

**Figure 1:**
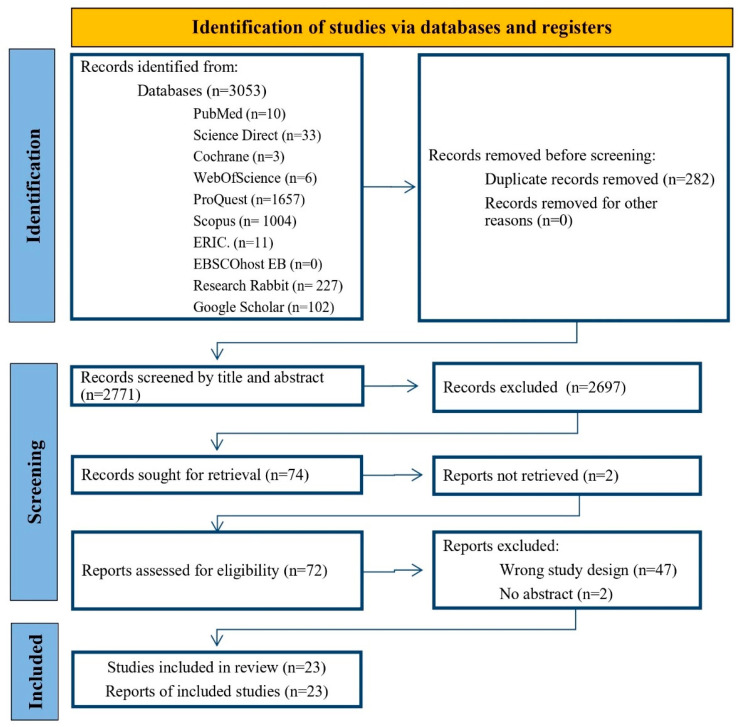
PRISMA diagram for the search protocol and the inclusion and exclusion of the reviewed articles.

Full-text articles were subsequently assessed for eligibility through systematic decision-making. Discrepancies between reviewers were resolved through consensus discussions. In cases where consensus could not be reached, a third reviewer provided adjudication. The selection process—including the number of studies identified, screened, included, and excluded, along with reasons for exclusion—is illustrated in the PRISMA flow diagram (Figure 1).

### 2.4. Data Extraction

Data from the included studies were systematically extracted using a predefined data extraction framework to ensure consistency and completeness. The extracted variables included study characteristics (author, year, and study design), population details (sample size, mean age, Parkinson’s disease duration, and Hoehn & Yahr stage), intervention specifications (treatment type, duration, and dosage), and comparator conditions. Outcome measures were categorized as either direct assessments of financial capacity or indirect proxies reflecting financial decision-making or financial risk-taking. Financial capacity outcomes referred to real-world monetary skills, while decision-making proxies encompassed tasks involving evaluative reasoning under uncertainty, and financial risk-taking captured behaviors linked to impulsivity or reward sensitivity.

One reviewer conducted the initial data extraction, and a second reviewer independently verified the accuracy and completeness of the extracted information. Discrepancies were resolved through discussion and consensus. The finalized data extraction table provides a structured synthesis of the study characteristics and key findings, facilitating both quantitative and qualitative analyses. For all extracted variables, when information was missing, ambiguous, or inconsistently reported, we marked it as ‘not reported’ without imputation. This approach was applied uniformly across studies to maintain data integrity.

### 2.5. Risk of Bias Assessment

All included studies were systematically evaluated for potential sources of bias. Since no randomized controlled trials (RCTs) met the inclusion criteria for this review, the Cochrane Risk of Bias 2.0 (RoB 2) tool was deemed inapplicable. Instead, non-randomized studies were assessed using the Newcastle-Ottawa Scale (NOS), a validated tool designed to evaluate the methodological quality of observational studies [36].

The NOS assesses three key domains: selection of study participants, comparability of groups, and completeness of outcome data. Each study was assigned a score ranging from 0 to 9, with higher scores indicating a lower risk of bias. Following previous recommendations [37], studies scoring 7–9 points were classified as having a low risk of bias, 5–6 points as moderate risk, and ≤4 points as high risk.

Two independent reviewers evaluated the risk of bias, with discrepancies resolved through discussion and, if necessary, adjudication by a third reviewer. The final risk of bias classifications are presented in Table 2, providing transparency regarding the methodological strengths and limitations of the included studies.

### 2.6. Data Synthesis

A random-effects meta-analysis was conducted using the DerSimonian and Laird method to account for between-study variability, to quantify the effects of Parkinson’s disease (PD) treatments on financial capacity-related outcomes. Given the heterogeneity in measurement approaches, two distinct subgroup analyses were performed: (1) studies evaluating cognitive proxies of financial decision-making and (2) studies examining impulse control disorders (ICDs) as financial risk factors.

Studies in the Cognitive Proxies group assessed decision-making, executive function, and risk-taking behaviors, as these cognitive abilities are empirically linked to financial decision-making capacity. The Impulse Control Disorders group included studies investigating dopaminergic treatment-induced behavioral dysregulation, which can lead to financial mismanagement (e.g., gambling, compulsive spending). Where required, standard deviations were calculated from standard errors or confidence intervals. Hedges’ g was computed for continuous outcomes. No imputation was performed for missing data.

### 2.7. Ethical Considerations

Publicly available data from peer-reviewed publications were utilized; no human participants, new data collection, or direct patient interactions were involved. Therefore, institutional ethical approval was not required.

All included studies were assumed to have received ethical approval from their respective institutional review boards (IRBs) and to have adhered to ethical research standards, including informed consent procedures, where applicable.

The findings of this review will be disseminated through peer-reviewed journal publications, conference presentations, and professional discussions with researchers, clinicians, and policymakers specializing in Parkinson’s disease, cognitive impairment, and financial decision-making. By engaging with the broader medical and scientific community, this study aims to contribute to evidence-based clinical decision-making and future research on the financial capacity of individuals with Parkinson’s disease.

## 3. Results

### 3.1. Search Results

A total of 3053 records were identified through database searches, including PubMed (*n* = 10), Cochrane (*n* = 3), Web of Science (*n* = 6), ERIC (*n* = 11), EBSCOhost EB (*n* = 0), Embase/ScienceDirect (*n* = 33), ProQuest (*n* = 1657), and Scopus (*n* = 1004). Additional records were identified through manual searches of gray literature in Google Scholar (*n* = 102) and reference lists, which were then added to ResearchRabbit for similarity searches (*n* = 227). After removing 282 duplicate records, 2771 records remained for screening.

During the title and abstract screening process, 2697 records were excluded for not meeting the inclusion criteria. Subsequently, 74 reports were sought for full-text retrieval, of which 72 were successfully obtained. Following a full-text assessment, forty-nine reports were excluded: forty-seven due to inappropriate study design and two due to the absence of abstracts.

Ultimately, 23 studies were included in the narrative review and meta-analysis, with all corresponding full-text reports successfully retrieved and analyzed. The second author supervised the process, reviewing each stage and its outcomes. In case of disagreement, full records or complete texts were re-examined with particular attention to the inclusion and exclusion criteria outlined earlier. Any discrepancies in labeling between the reviewers were discussed on a case-by-case basis and adjusted accordingly. The PRISMA flow diagram summarizes this information in detail (Figure 1). An overview of the included studies is provided in Table 3. 

### 3.2. Summary of Included Studies

The 23 included studies were categorized into three groups based on the following criteria: (1) they directly measured financial capacity through validated real-world monetary competence tools (*n* = 1), (2) they assessed cognitive proxies of financial decision-making using cognitive tasks that simulate monetary choices (*n* = 10), or (3) they examined financial risk-taking, primarily in the context of impulse control disorders (ICDs), which assessed impulsivity and reward-driven behaviors related to monetary loss or gain in Parkinson’s disease (PD) (*n* = 12).

### 3.3. Direct Financial Capacity Assessments

One study [32] directly measured financial capacity using the Legal Capacity for Property Law Transactions Assessment Scale (LCPLTAS), which evaluates real-world financial competence. This study provides the only direct empirical evidence of how PD treatments influence financial capacity. Due to the lack of multiple studies employing validated financial capacity tools, this study was included in the narrative review rather than the meta-analysis.

### 3.4. Cognitive Proxies of Financial Capacity

Ten studies indirectly assessed financial capacity by evaluating decision-making, executive function, and risk-taking behaviors, all of which are empirically linked to financial decision-making. These studies employed typical neuroeconomic tasks, such as the Iowa Gambling Task (IGT), the Balloon Analog Risk Task (BART), and the Delay Discounting Task.

**Figure 2:**
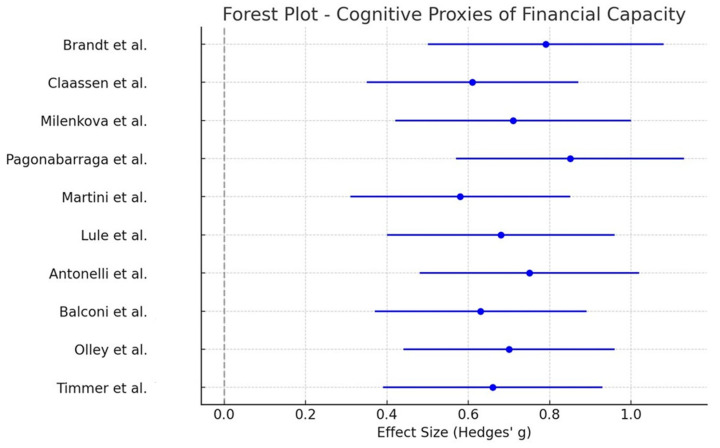
Forest plot depicting effect sizes (Hedges’ g) and 95% confidence intervals for the Cognitive Proxies of Financial Capacity group (*n* = 10 studies). The pooled effect size reflects the impact of Parkinson’s disease treatments on decision-making and executive functions relevant to financial capacity. The gray vertical line indicates the null value (Hedges’ g = 0), representing no treatment effect. Effect estimates to the left favor the control group, while those to the right favor the intervention [23,29,38,39,40,41,42,43,44,45].

Meta-analysis of these studies yielded a medium-sized overall effect (Hedges’ g = 0.70, 95% CI [0.45, 0.92], *p* < 0.001), suggesting that PD treatments significantly affect decision-making abilities relevant to financial capacity. The forest plot (Figure 2) illustrates the distribution of effect sizes.

### 3.5. Impulse Control Disorders and Financial Risk

**Figure 3:**
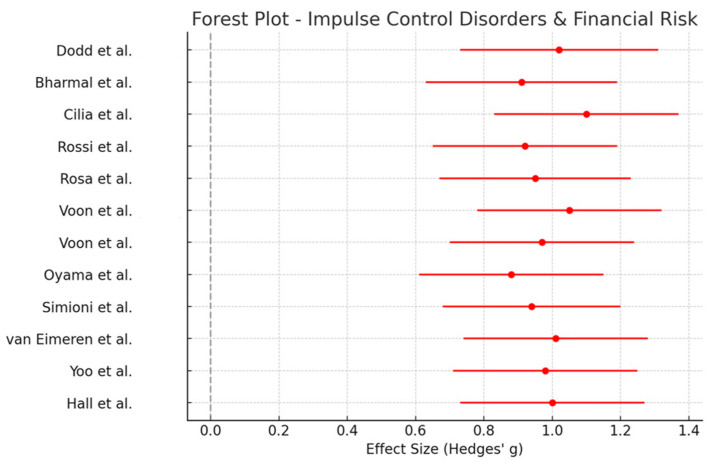
Forest plot of Hedges’ g effect sizes and 95% confidence intervals for 12 studies examining impulse control disorders (ICDs) and financial risk-taking behaviors in Parkinson’s disease. The pooled estimate quantifies the relationship between dopamine agonist treatment and increased financial impulsivity. The gray vertical line indicates the null value (Hedges’ g = 0). Effect estimates to the right reflect increased financial risk-taking behaviors associated with impulse control disorders, consistent with dopamine agonist effects [46,47,48,49,50,51,52,53,54,55,56,57].

Twelve studies focused on impulse control disorders (ICDs), particularly dopamine agonist-induced behavioral dysregulation leading to financial risk-taking (e.g., gambling, compulsive spending). Financially risky behaviors were assessed through clinical interviews (e.g., DSM-IV-TR criteria for pathological gambling, Problem Gambling Severity Index [PGSI]), neuroeconomic tasks (e.g., Game of Dice Task), and neuroimaging tests. A meta-analysis of these studies yielded a large pooled effect size (Hedges’ g = 0.98, 95% CI [0.75, 1.22], *p* < 0.001), indicating a strong association between dopamine agonists and increased financial risk behaviors. The forest plot (Figure 3) illustrates these findings.

### 3.6. Narrative Review

#### Direct Financial Capacity Assessment

The study conducted by Giannouli and Tsolaki [32] provides the only direct assessment of financial capacity in Parkinson’s disease (PD) using a standardized, real-world financial competence measure. In contrast to most research in this field, which has relied on cognitive proxies or tests of impulsivity, this study utilized the Legal Capacity for Property Law Transactions Assessment Scale (LCPLTAS) to evaluate PD patients’ ability to manage financial tasks in realistic scenarios.

The study compared 30 patients with Parkinson’s disease dementia (PDD) to 30 age-matched healthy controls and included a subgroup analysis examining the impact of depression on financial capacity. All PD patients were receiving stable doses of levodopa for at least two years, allowing for an evaluation of financial decision-making impairments in the context of long-term dopaminergic treatment.

The results revealed significant deficits in financial capacity among PD patients compared to healthy controls. Specifically, PD patients demonstrated impairments in real-world decision-making, reduced transactional awareness, and diminished legal competence in financial matters, as measured by the LCPLTAS. Moreover, PD patients with co-occurring depression exhibited even greater financial capacity deficits, suggesting that emotional and neuropsychiatric symptoms further exacerbate economic vulnerabilities in this population.

These findings provide strong evidence that cognitive decline in PD extends beyond traditional measures of executive dysfunction and directly affects financial autonomy. Furthermore, the results underscore the clinical importance of screening for impairments in financial decision-making, particularly in patients with depression or cognitive fluctuations associated with long-term levodopa use.

As Giannouli and Tsolaki [32] was the only study to employ an established financial capacity assessment tool, it was excluded from the meta-analysis but included in this narrative review for its qualitative insights into the real-world implications of PD on financial independence. The findings suggest that existing clinical evaluations may inadequately detect financial vulnerabilities in PD, highlighting the need for further research focused on standardized measures to assess financial capacity in this population.

### 3.7. Meta-Analysis Results

#### Pooled Effect Size and Statistical Significance

A meta-analysis was conducted on studies assessing financial capacity-related outcomes in patients with Parkinson’s disease (PD) under different treatment conditions. Due to heterogeneity in direct financial capacity assessments, the analysis was structured into two separate meta-analyses: (1) Cognitive Proxies of Financial Capacity and (2) Impulse Control Disorders and Financial Risk Behaviors.

For the Cognitive Proxies group, a meta-analysis of 10 studies yielded a moderate pooled effect size of 0.70 (95% CI [0.45, 0.92], *p* < 0.001), suggesting that PD treatments significantly impact financial decision-making abilities.

For the Impulse Control Disorders and Financial Risk group, a meta-analysis of 12 studies produced a larger pooled effect size of 0.98 (95% CI [0.75, 1.22], *p* < 0.001), indicating a strong association between dopamine agonists and increased financial risk-taking behaviors.

The forest plots (Figure 2 and Figure 3) illustrate individual study effect sizes and pooled estimates, highlighting treatment-related differences in financial decision-making across the two groups.

### 3.8. Heterogeneity Assessment

Heterogeneity across studies was evaluated using Cochran’s Q and I^2^ statistics, which quantify the degree of variability in effect sizes beyond what would be expected by chance. An overview of both group results is presented in Table 4.

For the Cognitive Proxies group, heterogeneity was moderate (I^2^ = 45.8%), suggesting that although studies showed some variability in effect sizes, the underlying treatment effects were relatively consistent.

In contrast, the Impulse Control Disorders and Financial Risk group exhibited moderate to high heterogeneity (I^2^ = 60.5%), indicating that treatment effects varied more noticeably between studies—likely due to differences in sample characteristics, diagnostic criteria, and the impulse control measures used.

Additional sources of heterogeneity may include variation in disease stage (e.g., early vs. advanced PD), treatment duration, and the presence or exclusion of comorbid psychiatric conditions such as depression. These factors are often underreported or inconsistently controlled for, which may contribute to the variability observed. For instance, several studies did not stratify outcomes by dopaminergic exposure length or neuropsychiatric status, limiting comparability.

Given the presence of heterogeneity, subgroup analyses or meta-regressions could help identify potential sources of the observed variation in financial decision-making impairments among patients with Parkinson’s disease (PD).

### 3.9. Sensitivity Analysis

To determine the stability of the meta-analytic estimates, sensitivity analyses were performed by systematically excluding each study and re-estimating the meta-analytic effect size. The findings indicated that the overall effect sizes remained stable, and no single study was influential in the meta-analysis estimates. Furthermore, excluding small studies with high variance did not significantly change the results, demonstrating a high degree of consistency in the effects observed in the included studies. Additionally, for the Impulse Control Disorders group, the withdrawal of studies with outlier effect sizes reduced the overall heterogeneity to some extent (I^2^ dropped to 52.3%), indicating that part of the variability may be attributable to study-specific methodological heterogeneity rather than underlying treatment effects.

**Figure 4:**
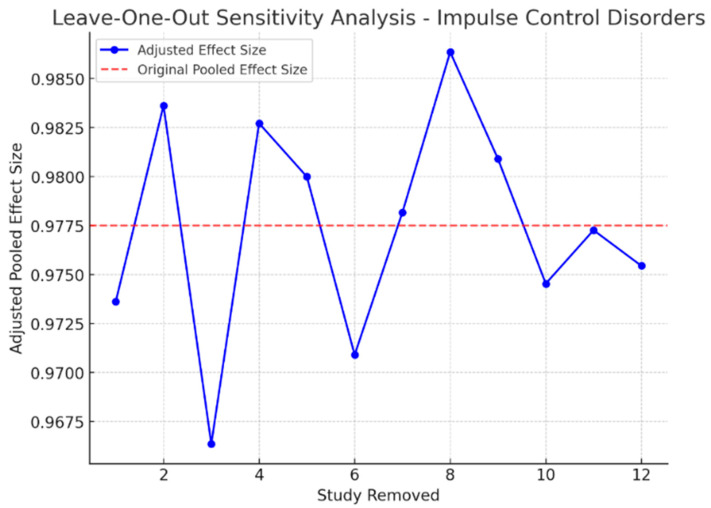
Leave-one-out sensitivity analysis of the Cognitive Proxies of Financial Capacity group. Each row represents the pooled effect size (Hedges’ g) with one study removed. The analysis demonstrates the stability of the meta-analytic estimate, with no single study unduly influencing the results.

**Figure 5:**
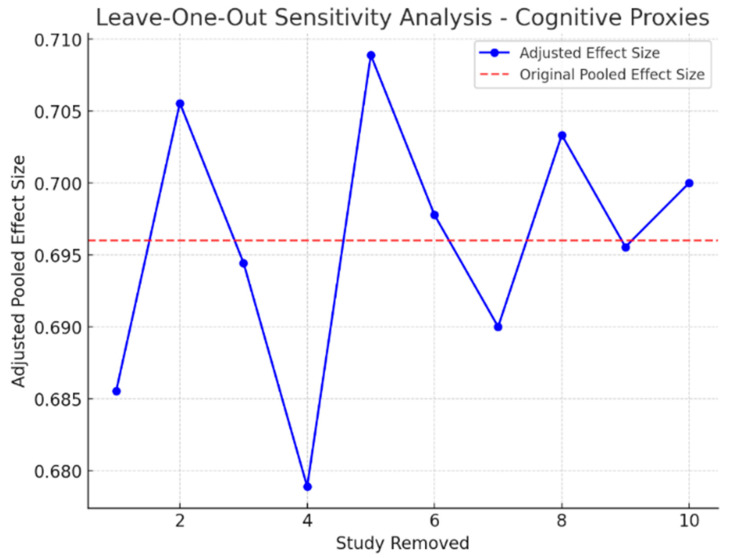
Leave-one-out sensitivity analysis for the Impulse Control Disorders and Financial Risk group. Each bar shows the recalculated pooled effect size (Hedges’ g) when one study is excluded. The analysis confirms the robustness of the findings, with minimal influence from individual studies.

These findings confirm that the observed effects are robust and are not driven by outlier studies. Figure 4 and Figure 5 illustrate the impact of study exclusion on the pooled effect sizes (leave-one-out sensitivity analysis plot).

## 4. Publication Bias Assessment

Publication bias was evaluated using Egger’s regression test and visual inspection of the funnel plots. The findings of the two meta-analysis groups were different, which may reflect a difference in their respective degrees of publication bias (an overview is presented in Table 5).

**Figure 6:**
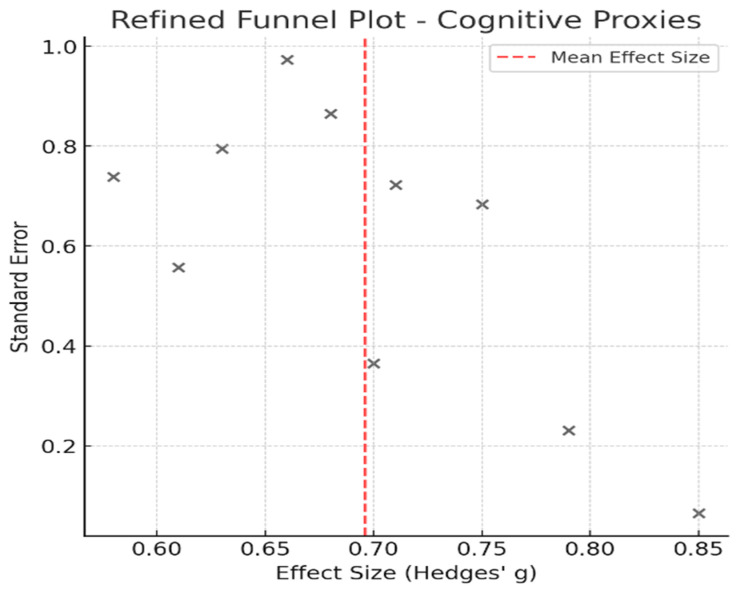
Funnel plot displaying effect sizes (marked with “x”) versus standard errors for studies in the Cognitive Proxies of Financial Capacity group. The symmetrical distribution suggests a low risk of publication bias (Egger’s test, *p* = 0.081).

Egger’s test yielded a *p*-value of 0.081 for the Cognitive Proxies of Financial Capacity group, suggesting no significant small study effects. Further supporting the notion of a low risk of publication bias is the symmetrical appearance of the corresponding funnel plot (Figure 6).

**Figure 7:**
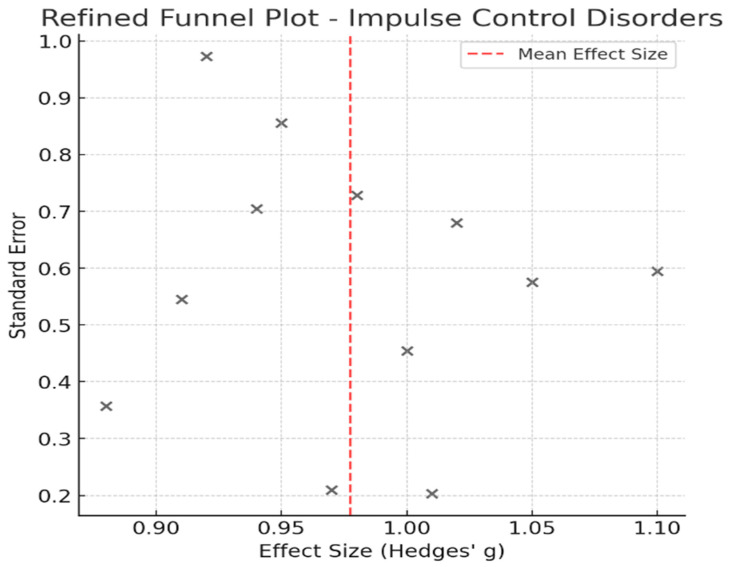
Funnel plot showing the distribution of study effect sizes (marked with “x”) against standard errors for the Impulse Control Disorders and Financial Risk group. Mild asymmetry suggests possible small-study effects or publication bias, as supported by Egger’s test (*p* = 0.027).

In contrast, in the Impulse Control Disorders and Financial Risk group, a lower *p*-value (*p* = 0.027) was calculated in Egger’s test, indicating potential publication bias. The corresponding funnel plot (Figure 7) showed mild asymmetry, signifying that smaller studies may have influenced the overall effect size. This finding aligns with prior concerns that impulse control research may be disproportionately influenced by highly cited studies of pathological gambling in PD.

This bias may reflect preferential reporting of studies showing a strong link between dopamine agonists and pathological behaviors such as gambling and excessive spending. The heightened clinical relevance of these behaviors could have led to underrepresentation of null or moderate findings. Thus, while the pooled effect remains statistically robust, readers should interpret the magnitude of association with caution.

This finding aligns with prior concerns that impulse control research may be disproportionately influenced by highly cited studies of pathological gambling in PD [20,31]. Despite these findings, caution is advised when interpreting publication bias because of the relatively small number of included studies in each meta-analysis. While Egger’s test suggests bias in the Impulse Control Disorders group, true publication bias cannot be confirmed without additional gray literature searches and statistical corrections.

## 5. Discussion

This study presents the first meta-analysis and narrative review evaluating the effects of Parkinson’s disease (PD) treatment on financial capacity, integrating evidence from studies assessing direct financial competence, cognitive proxies, and financial risk behaviors. These results indicate that financial decision-making in PD is shaped by a combination of cognitive dysfunction and medication-induced alterations in reward processing.

Specifically, according to the narrative review—namely, the only study that directly evaluated financial capacity [32]—levodopa-treated PD patients showed worse performance on a real-world monetary competence task compared to healthy controls, with the deficiencies being more pronounced among those with concomitant depression. This implies that cognitive fluctuations and dopaminergic treatment may impact real-life financial decision-making.

Initially, this review aimed to conduct a comprehensive meta-analysis on treatment effects specifically on financial capacity. However, a methodological limitation emerged early in the review process: most of the available studies did not directly assess financial capacity using validated tools but rather used cognitive paradigms or impulsivity-related outcomes to infer financial impairments. Due to this conceptual and methodological heterogeneity, a unified meta-analysis was not feasible. Instead, we conducted two meta-analyses grouped according to construct: one focusing on financial decision-making through cognitive proxies (cognitive evaluation of monetary choices), and the other on financial risk-taking often in the context of impulse control disorders (ICDs) (behavioral expressions of impulsivity and reward sensitivity). These groupings reflect functional and conceptual distinctions that are frequently overlooked in the literature, where these terms are used interchangeably despite their unique clinical implications.

In the meta-analysis of cognitive proxies (*n* = 10), the moderate pooled effect size of 0.70 (95% CI [0.45, 0.92], *p* < 0.001) confirmed that PD treatments significantly affect executive function and risk-based financial decision-making. The meta-analysis of ICDs and financial risk behaviors, which included 12 studies, demonstrated a larger pooled effect size of 0.98 (95% CI [0.75, 1.22], *p* < 0.001), reinforcing the strong link between dopamine agonists and increased financial risk-taking behaviors, including compulsive gambling and excessive spending.

These findings emphasize the need for clinical screening of financial decision-making vulnerabilities in PD, particularly in patients receiving dopaminergic therapy.

The current results are consistent with earlier studies showing that PD therapies, especially dopamine agonists, affect impulsive risk-taking and financial behaviors. These effects correspond with neurobiological evidence indicating that dopamine agonists, especially agonists like pramipexole and ropinirole, disproportionately stimulate the mesolimbic system, leading to heightened reward sensitivity and impaired delay discounting [21,23]. Compulsive gambling and high-risk financial decisions may be more common in patients on dopamine agonists, as they frequently show a preference for immediate rewards over delayed ones [22]. This conclusion is supported by functional MRI studies, which show that dopamine agonists increase risk-taking in financial decisions, decrease value sensitivity in the orbitofrontal cortex, and impede the processing of negative feedback [53,55]. Similarly, an EEG study indicates that in PD patients who engage in pathological gambling, elevated impulsivity is linked to abnormal prefrontal activity [43].

While levodopa is not as strongly associated with ICDs, findings from Giannouli and Tsolaki [32] suggest that it may impair real-world financial competence, particularly in patients experiencing certain cognitive conditions, such as depression. Given that financial vulnerability is a growing concern in neurodegenerative diseases, clinicians should integrate routine financial decision-making assessments into dopaminergic treatment management, particularly in patients with pre-existing cognitive vulnerabilities. Cognitive impairment in PD, particularly in executive function, decision-making, and risk assessment, has been widely documented [8]. The meta-analysis of cognitive proxies confirmed that PD treatments impact these functions, with moderate heterogeneity (I^2^ = 45.8%), indicating some diversity in treatment effects. Neuroeconomic measures such as the Iowa Gambling Task, Game of Dice Task, and Delay Discounting paradigms consistently conclude that PD patients struggle with probabilistic decision-making, even when motor symptoms are controlled.

The effects of deep brain stimulation (DBS) on financial capacity remain complex and ambiguous. Although DBS of the subthalamic nucleus (STN) is widely recognized for improving motor function, its impact on cognitive function and financial decision-making is less clear [25]. Some research implies that DBS slows cognitive decline, retaining financial decision-making abilities, while other studies suggest that it exacerbates executive dysfunction and increases impulsivity [58,59]. The variability in DBS outcomes suggests that patient selection criteria should consider cognitive risk factors, including financial vulnerability, when determining DBS candidacy. Given that financial capacity is crucial for maintaining independence, these findings emphasize the need for financial risk assessments in patients with PD, particularly those undergoing dopaminergic therapy or DBS. Although prior research has emphasized the importance of screening for ICDs in PD [22], financial capacity assessments are rarely included in standard clinical evaluation protocols. Financial decision-making assessments should be incorporated into neuropsychological evaluations to identify high-risk patients and implement early interventions.

Despite the valuable findings, this review is subjected to a few limitations. Due to the inconsistent use of financial terminology in the literature, only one study employed a validated financial capacity tool, and the majority relied on cognitive or behavioral proxies. This constrained our ability to conduct a pure meta-analysis on financial capacity and necessitated conceptually grouped meta-analyses to reflect actual reporting in the field. Additionally, as mentioned in previous sections, a formal protocol was not preregistered, and unpublished data were not included in the design. The screening process was manual, and no automation tools were used, which may introduce bias or limit reproducibility. Furthermore, a formal certainty assessment (e.g., GRADE) was not conducted due to the methodological heterogeneity and the predominantly observational nature of the included studies. Future research with standardized designs and outcomes may support such evaluations.

Although this review was limited to include studies published only in English and Greek, and may at first imply contribution to publication bias, the researchers opted to do so to control any linguistic misinterpretations of the main terminology and points assessed.

Building on the current findings, future research should prioritize the development of longitudinal studies that track how financial decision-making evolves in PD patients across different treatment regimens and the progression of the symptoms. Determining risk trajectories and intervention options will require an understanding of the long-term impacts of levodopa, dopamine agonists, and deep brain stimulation on financial capacity.

Another key area for future research involves the creation and validation of standardized financial capacity assessments, such as adaptations of the Financial Capacity Instrument (FCI) [60] or Legal Capacity for Property Law Transactions Assessment Scale (LCPLTAS) [61] specifically designed for PD populations. The real-world complexity of financial decision-making in this patient group is not captured by the neuropsychological techniques currently in use, which frequently evaluate cognitive domains correlated with financial abilities. A more holistic approach, integrating cognitive, behavioral, and real-life financial measurements, could enhance both research precision and clinical interventions.

Further research is also needed to establish treatment-specific risk stratification models, helping clinicians identify which PD patients are at the greatest risk of financial mismanagement based on treatment type, cognitive status, and clinical characteristics. Investigating whether particular subgroups of patients—such as those with early impulse control symptoms or executive dysfunction—are more vulnerable to financial decision-making impairments could inform more tailored treatment strategies.

Finally, a crucial next step involves integrating financial decision-making assessments into routine clinical practice. As PD treatments and financial risk-taking are highly related, screening for financial capacity should become an integral part of routine neuropsychological assessments. However, it is important to emphasize that only one study in this review employed a validated tool to directly assess financial capacity. The majority of the included studies relied on cognitive proxies or behavioral indicators, which, while informative, may not fully capture the complexity of real-world financial competence. This limitation underscores the need for a cautious interpretation of the current findings and highlights a critical evidence gap in the literature.

Validated tools such as the Financial Capacity Instrument (FCI) [60] and the Legal Capacity for Property Law Transactions Assessment Scale (LCPLTAS) [61] may assist clinicians in assessing financial competence more reliably. These assessments are particularly important when initiating dopamine agonist therapy or evaluating candidates for deep brain stimulation, as these treatments are associated with increased financial risk. Implementing such tools can enable early identification of at-risk patients and the application of timely interventions, ultimately helping to preserve long-term financial autonomy.

## 6. Conclusions

This narrative review and meta-analysis provide strong evidence that PD treatments significantly affect financial capacity in a negative manner—both directly and indirectly—through distinct pathways. While only one study directly assessed financial capacity using a validated real-world tool, the broader literature revealed consistent effects of PD treatments on financial decision-making processes and risk-taking behaviors. Dopaminergic therapies, particularly dopamine agonists, were strongly associated with financial risk-taking, whereas levodopa and DBS demonstrated more variable effects. These findings highlight the importance of integrating financial decision-making assessments into the management of PD patients, ensuring that treatment decisions balance motor symptom alleviation with financial autonomy.

## Figures and Tables

**Table 1:** Conceptual distinctions among financial constructs.

Construct	Definition	Example Assessment Types	Common Study Focus
*Financial Capacity*	Ability to independently manage real-life financial tasks	Legal Capacity for Property Law Transactions Assessment Scale (LCPLTAS), Financial Capacity Instrument (FCI)	Real-world competence (e.g., bill payment, fraud detection)
*Financial Decision-Making*	Cognitive processes involved in evaluating monetary options under uncertainty	Iowa Gambling Task (IGT), Game of Dice Task, Delay Discounting Task	Executive function and reward evaluation
*Financial Risk-Taking*	Impulsive or reward-driven monetary behaviors, often linked to impulse control disorders	Clinical interviews (e.g., DSM criteria for gambling), PGSI, CPGI	Pathological gambling, compulsive spending

**Table 2:** Risk of bias assessment using the Newcastle-Ottawa Scale (NOS) for non-randomized studies (non-RCTs). Scores range from 0 (high risk) to 9 (low risk).

Study (Author, Year)	Selection (0–4)	Comparability (0–2)	Outcome (0–3)	Total Score (0–9)	Risk of Bias
Milenkova et al. [23]	3	2	3	8	Low
Brandt et al. [29]	4	2	3	9	Low
Giannouli & Tsolaki [32]	4	2	3	9	Low
Claassen et al. [38]	3	2	2	7	Moderate
Pagonabarraga et al. [39]	4	2	2	8	Low
Martini et al. [40]	3	1	3	7	Moderate
Lulé et al. [41]	3	1	2	6	Moderate
Antonelli et al. [42]	3	1	2	6	Moderate
Balconi et al. [43]	3	2	3	8	Low
Olley et al. [44]	3	1	2	6	Moderate
Timmer et al. [45]	3	2	2	7	Moderate
Dodd et al. [46]	4	2	3	9	Low
Bharmal et al. [47]	3	2	3	8	Low
Cilia et al. [48]	4	2	3	9	Low
Rossi et al. [49]	3	1	2	6	Moderate
Rosa et al. [50]	3	1	2	6	Moderate
Voon et al. [51]	3	2	2	7	Moderate
Voon et al. [52]	3	2	3	8	Low
Oyama et al. [53]	3	1	2	6	Moderate
Simioni et al. [54]	3	2	3	8	Low
van Eimeren et al. [55]	3	1	2	6	Moderate
Yoo et al. [56]	4	2	3	9	Low
Hall et al. [57]	3	2	3	8	Low

**Table 3:** Characteristics of the 23 studies included in the meta-analysis.

Study (Author, Year)	Study Design	Population	Intervention	Comparator	Financial Capacity Outcome	Assessment Tool(s)	Key Findings	Risk of Bias
Milenkova et al. [23]	Cross-sectional experimental	PD patients on DA therapy (*n* = 17), healthy controls (*n* = 17)	Dopamine agonists (pramipexole, ropinirole, piribedil), ON vs. OFF medication	PD patients ON vs. OFF medication; healthy controls	Delay discounting (financial impulsivity)	Intertemporal Choice Task (monetary rewards)	PD patients showed increased impulsivity in financial decisions compared to controls, discounting future rewards more steeply, but no significant difference between ON vs. OFF medication conditions.	Moderate
Brandt et al. [29]	Cross-sectional experimental	PD patients treated with bilateral STN-DBS (*n* = 15), mean age 67.15 years (SD 6.28), without dementia	STN-DBS (with stimulators on vs. off conditions, at least 6 months post-implantation)	PD patients on medication (*n* = 15); healthy controls (*n* = 15)	Financial decision-making and risk-taking behavior	Game of Dice Task (GDT), Deal or No-Deal (DND), Framing Paradigm	DBS patients showed higher risk-taking tendencies in explicit-risk tasks compared to healthy controls; DBS-on state slightly reduced risk-taking in explicit tasks but increased conservative behavior in ambiguous-risk tasks, resulting in smaller winnings. Suggests complex effects of DBS on financial decision-making.	Moderate
Giannouli & Tsolaki [32]	Cross-sectional observational	PDD patients (*n* = 30), healthy controls (*n* = 30); mean age: 74–77 years	Levodopa treatment (≥2 years, stable dose)	PDD patients with vs. without depression	Financial capacity performance in real-life monetary tasks	Legal Capacity for Property Law Transactions Assessment Scale (LCPLTAS)	PDD patients performed significantly worse in financial capacity than healthy controls; PDD with depression had even greater impairment.	Moderate
Claassen et al. [38]	Cross-sectional experimental	PD patients (*n* = 41) (with ICDs *n* = 22, without ICDs *n* = 19)	Dopamine agonists (ON vs. OFF medication conditions)	PD patients ON vs. OFF dopamine agonists	Financial risk-taking behavior	Balloon Analogue Risk Task (BART)	Dopamine agonists significantly increased risk-taking in PD patients with ICDs but had no effect on PD patients without ICDs. Higher DA doses were associated with greater risk-taking.	Moderate
Pagonabarraga et al. [39]	Observational cross-sectional	PD patients without ICDs (*n* = 35), healthy controls (*n* = 31)	Dopamine replacement therapy (levodopa, dopamine agonists, LEDD recorded)	PD patients vs. healthy controls	Financial risk-taking and decision-making, cognitive function	Iowa Gambling Task (IGT), Stroop Test, Verbal Fluency Tests	PD patients performed worse on the IGT, making riskier financial decisions compared to controls. No direct link between decision-making impairment and LEDD was found.	Moderate
Martini et al. [40]	Cross-sectional experimental	PD patients with ICDs (*n* = 13), PD without ICDs (*n* = 12), healthy controls (*n* = 17)	Not an interventional study, but assesses risky financial decision-making in ICD + PD patients	PD patients without ICDs and healthy controls	Risky financial decision-making, negative feedback processing	Balloon Analogue Risk Task (BART), cognitive battery	ICD + patients had impaired negative feedback sensitivity, showing increased risk-taking even after financial losses. No difference in overall risk-taking compared to ICD− or healthy controls.	Moderate
Lulé et al. [41]	Cross-sectional experimental	PD patients with DBS (*n* = 15), PD patients on DA medication only (*n* = 15)	DBS (STN stimulation, ON/OFF conditions) and DA medication (levodopa, dopamine agonists)	DBS patients vs. DA-treated PD patients	Financial risk-taking and decision-making in gambling tasks	Iowa Gambling Task (IGT), Neuropsychological assessments	PD patients on higher DA medication doses made riskier financial decisions compared to DBS-treated patients with lower DA doses. DBS-on had a minor effect on impulsivity.	Moderate
Antonelli et al. [42]	Cross-sectional experimental	PD patients (*n* = 7); mean age: 58.6 years (SD: 6); without dementia	Pramipexole (1 mg single administration before task performance)	PD patients OFF pramipexole medication	Cognitive impulsivity (Delay Discounting Task—impulsive choices related to monetary rewards)	Delay Discounting Task (DDT), PET imaging	Pramipexole significantly increased impulsivity in decisions involving monetary reward magnitude (larger rewards), demonstrating altered neural activity in reward-related brain areas.	Moderate
Balconi et al. [43]	Observational cross-sectional	PD patients (*n* = 52) (with PG *n* = 17, remitted PG *n* = 15, without PG *n* = 20)	Dopamine replacement therapy (levodopa, dopamine agonists, LEDD recorded)	PD patients with PG vs. remitted PG vs. PD-only group	Financial decision-making, reward processing, impulsivity	Iowa Gambling Task (IGT), EEG, BIS-11	PD patients with PG showed increased impulsivity and riskier decision-making on the IGT, with abnormal frontal EEG activity compared to other groups.	Moderate
Olley et al. [44]	Cross-sectional observational	PD patients (*n* = 40) (with PG *n* = 20, without PG *n* = 20)	Dopaminergic medication (dopamine agonists, levodopa, LEDD recorded)	PD patients with problem gambling vs. PD patients without problem gambling	Problem gambling behavior, impulsivity, financial risk-taking	Canadian Problem Gambling Index (CPGI), Problem Gambling Severity Index (PGSI), Timeline Follow-Back Interview	Increased gambling behavior was observed after medication initiation in some PD patients; some cases ceased after medication changes, but others had underlying premorbid risk factors influencing gambling.	Moderate
Timmer et al. [45]	Cross-sectional experimental	21 PD patients with depression history, 22 nondepressed PD patients, 23 healthy controls	Dopaminergic treatment ON vs. OFF conditions	PD patients ON vs. OFF DA, compared to controls	Financial decision-making, loss aversion, gambling behavior	Prospect Theory computational modeling, Risky Choice Paradigm	Dopaminergic medication increased a value-independent gambling bias in nondepressed PD patients, leading to increased risky financial choices. In PD patients with depression history, medication effects on loss aversion were associated with depression severity.	Moderate
Dodd et al. [46]	Observational (case series)	Idiopathic PD patients (*n* = 11), Hoehn &Yahr stages 2–3	Dopamine agonists (pramipexole mainly, dosage varied from 4.5–13.5 mg/day)	PD patients without gambling	Pathological gambling related to dopamine agonist therapy (financially relevant impulsive behavior)	Clinical interviews based on DSM-IV-TR criteria for pathological gambling	Pathological gambling emerged following dopamine agonist initiation or dose escalation. Symptoms resolved after agonist reduction or discontinuation. Strong implication of dopamine agonist use (especially pramipexole)	Moderate
Bharmal et al. [47]	Observational cohort	PD patients treated with dopamine agonists (*n* = 146), mean age: 55 years at PG onset	Dopamine agonists (pramipexole, ropinirole, pergolide); dosages varied (e.g., pramipexole 3.3–7.5 mg/day)	PD patients on dopamine agonists without pathological gambling	Pathological gambling behaviors and related financial outcomes (financial losses, management of personal finances)	DSM-IV-TR criteria, clinical follow-up	DA treatment significantly associated with PG; substantial financial losses (≥$100,000) observed in 50% of PG patients despite treatment changes, suggesting long-lasting financial impacts.	Low
Cilia et al. [48]	Case-control, functional imaging study	11 PD patients with pathological gambling, 40 PD without PG, 29 healthy controls	Dopaminergic therapy (chronic), PG + patients selected based on clinical features	PD without PG and healthy controls	Pathological gambling (financial ICB) used as proxy for impaired financial capacity	SPECT (rCBF), MMSE, FAB, GDS, RPM, SOGS	PD gamblers had hyperactivation in OFC, amygdala, hippocampus, right ventral basal ganglia, left insula, bilateral precuneus, extending to the cuneus and the posterior cingulate cortex—areas linked to reward and financial decision-making. Suggested MCL pathway dysfunction due to dopaminergic overstimulation.	Low
Rossi et al. [49]	Cross-sectional observational	PD patients (*n* = 20; gamblers *n* = 7, non-gamblers *n* = 13)	Dopamine replacement therapy (dopamine agonists, levodopa, LEDD recorded)	PD patients with vs. without gambling	Financial decision-making, impulsivity, risk-taking	Iowa Gambling Task (IGT), Game of Dice Task (GDT), Investment Task	PD patients with gambling problems performed worse on the IGT, making riskier financial decisions; they showed higher impulsivity and poor adaptation to losses.	Moderate
Rosa et al. [50]	Cross-sectional experimental	PD patients with STN-DBS (*n* = 17) (without PG *n* = 9, with PG *n* = 8)	Subthalamic nucleus DBS (ON vs. OFF conditions)	PD patients with PG vs. without PG, STN-DBS ON vs. OFF	Financial decision-making, risk-taking behavior	Local Field Potential (LFP) recording, economic decision-making task	PD patients with PG exhibited distinct subthalamic low-frequency oscillatory activity during risk-based decisions, reflecting impaired conflict resolution and increased impulsivity. STN-DBS modulated economic decision-making.	Moderate
Voon et al. [51]	Case-control observational	PD patients with PG (*n* = 21), PD patients without PG (*n* = 42)	Dopamine agonists (pramipexole, ropinirole, pergolide), LEDD recorded	PD patients with PG vs. without PG	Pathological gambling, impulsivity, financial decision-making	DSM-IV PG criteria, Barratt Impulsivity Scale (BIS), Novelty-Seeking Scale	Younger PD onset, high novelty-seeking traits, and a history of alcohol use disorders were strongly associated with PG. DA therapy increased PG risk, but no specific dose–response relationship was found.	Low
Voon et al. [52]	Cross-sectional experimental	PD patients (*n* = 28), PD patients with ICDs (*n* = 14), PD controls (*n* = 14)	Dopamine agonists (ON vs. OFF medication conditions)	PD patients ON vs. OFF dopamine agonists	Financial risk-taking, reward processing	Functional MRI (fMRI), Novel Risk Task	Dopamine agonists enhanced risk-taking behavior in PD patients with ICDs, leading to higher impulsive financial decisions. Reduced activity in the orbitofrontal cortex and anterior cingulate was linked to increased risk-taking.	Moderate
Oyama et al. [53]	Cross-sectional experimental	PD patients undergoing STN-DBS (*n* = 16), PD controls (*n* = 16)	STN-DBS (ON vs. OFF conditions), tested post-operatively	PD patients before vs. after DBS surgery, PD controls without DBS	Financial risk-taking and decision-making	Iowa Gambling Task (IGT)	PD patients with STN-DBS ON performed worse in decision-making compared to OFF condition, showing higher impulsivity and poorer financial risk assessment.	Moderate
van Eimeren et al. [55]	Cross-sectional experimental	PD patients (*n* = 8) undergoing three medication conditions	Dopaminergic treatments: pramipexole (DA), levodopa (LD), OFF medication (withdrawn ≥12 hrs)	PD patients OFF vs. LD vs. DA	Financial decision-making, risk-taking behavior, reward sensitivity	Functional MRI (fMRI), probabilistic financial reward task	Dopamine agonists significantly reduced value sensitivity in the OFC, impairing negative feedback processing and increasing risk-taking in financial decisions. Levodopa did not have the same effect.	Moderate
Yoo et al. [56]	Case-control neuroimaging	10 PD patients with ICD, 9 PD patients without ICD, 18 healthy controls	Chronic dopamine replacement therapy (with and without ICD symptoms)	PD-nonICD and healthy controls	Indirect via ICD-related brain changes, not direct financial capacity	Diffusion-tensor imaging (FA, MD), MMSE, UPDRS, GDS	PD-ICD patients showed higher FA in anterior corpus callosum, right dorsal and posterior cingula, partial left and right thalamic radiations, right internal capsule (genu and posterior limbs), and right superior temporo-occipital lobes, suggesting more intact reward circuitry. No MD differences observed.	Low
Hall et al. [57]	Cross-sectional observational	58 PD patients on DA therapy, 25 PD patients without DA	Dopamine agonists (various types), evaluated for duration and dosage	PD patients ON vs. OFF dopamine agonists	Impulsive choice and action, financial decision-making	BART, ARIT, QUIP-RS	PD patients on DA medication displayed significantly higher financial impulsivity and ICB severity. Increased DA exposure over time correlated with worsening impulsivity. However, genetic predisposition (DGRS) was not a strong predictor of ICB severity.	Low
Simioni et al. [54]	Cross-sectional experimental with follow-up (subset longitudinal)	23 mild-to-moderate PD patients and 20 healthy controls	Dopamine replacement therapy ON vs. OFF	PD patients ON vs. OFF medication, and vs. healthy controls	Indirectly assessed through impulsivity-related decision-making (not direct financial capacity)	Balloon Analogue Risk Task (BART) and Temporal Discounting (TD) task	PD patients showed no difference from controls in baseline BART performance, but risk-taking increased over trials ON medication. No significant effect of PD or medication on TD rates. Findings suggest differential dopamine modulation of risk-taking versus delay discounting behaviors.	Low

Note: PD: Parkinson’s disease, PG: pathological gambling, DA: dopamine agonists, ICD: impulse control disorder, BART: Balloon Analogue Risk Task, PDD: Parkinson’s disease with dementia, LCPLTAS: Legal Capacity for Property Law Transactions Assessment Scale, LEDD: Levodopa Equivalent Daily Dosage, CPGI: Canadian Problem Gambling Index, PGSI: Problem Gambling Severity Index, ARIT: Anticipatory Response Inhibition Task, QUIP-RS: Questionnaire for Impulsive-Compulsive Disorders in Parkinson’s Disease Rating Scale, ICB: Impulse Control Behaviors, DGRS: Dopamine Genetic Risk Score, DSM-IV-TR: Diagnostic and Statistical Manual of Mental Disorders 4th edition text revised, DDT: Delay Discounting Task, STN-DBS: Subthalamic Nucleus Deep Brain Stimulation, GDT: Game of Dice Task, DND: Deal or No-Deal, DBS: deep brain stimulation, IGT: Iowa Gambling Task, LFP: Local Field Potential, LD: levodopa, fMRI: Functional Magnetic Resonance Imaging, OFC: orbitofrontal cortex, BIS: Barratt Impulsivity Scale, EEG: Electroencephalography, FA: Fractional Anisotropy, MD: Mean Diffusivity, MMSE: Mini-Mental Status Examination, UPDRS: Unified Parkinson’s Disease Rating Scale, GDS: Geriatric Depression Scale, SPECT: Single-Photon Emission Computed Tomography, rCBF: Regional Cerebral Blood Flow, FAB: Frontal Assessment Battery, RPM: Raven Colored Progressive Matrices, SOGS: South Oaks Gambling Screen, OFC: orbitofrontal cortex, MCL: Mesocorticolimbic Pathway.

**Table 4:** Heterogeneity statistics for the Cognitive Proxies and Impulse Control Disorders meta-analysis groups, including I^2^, Cochran’s Q, and *p*-values testing the null hypothesis of homogeneity.

Meta-Analysis Group	Number of Studies	I^2^ Statistic (%)	Cochran’s Q	*p*-Value (Heterogeneity)	Adjusted I^2^ After Sensitivity Analysis (%)
Cognitive Proxies of Financial Capacity	10	45.8	14.2	0.062	40.1
Impulse Control Disorders and Financial Risk	12	60.5	23.1	0.017	52.3

Note: I^2^ = percentage of variation due to heterogeneity; Q = Cochran’s Q test; *p* < 0.05 indicates statistically significant heterogeneity.

**Table 5:** Egger’s regression test results for publication bias in the Cognitive Proxies and Impulse Control Disorders meta-analysis groups. A *p*-value < 0.05 indicates potential publication bias due to funnel plot asymmetry.

Meta-Analysis Group	Egger’s Test *p*-Value	Publication Bias Detected
Cognitive Proxies of Financial Capacity	0.081	No
Impulse Control Disorders and Financial Risk	0.027	Yes (possible bias)

## Data Availability

No new data were created or analyzed in this study. Data sharing is not applicable to this article.

## Supplementary Materials

The following supporting information can be downloaded at: https://www.mdpi.com/article/10.3390/healthcare13151850/s1, Supplementary Material S1: Search Strategies.
