# Supplementary material for: The Cognitive Cost of Motor Control: A Systematic Review and Meta-Analysis of Parkinson’s Disease Treatments and Financial Decision-Making
Source: Healthcare (Basel). 2025 Jul 29;13(15):1850. doi: 10.3390/healthcare13151850 (PMC12346847; doi:10.3390/healthcare13151850)
Supplement: Supplementary file 1 [file healthcare-13-01850-s001.zip › healthcare-3680041-supplementary.pdf]

Supplementary Material S1: Search Strategies

The following search strategies were used to identify studies related to Parkinson's disease (PD) treatments and financial capacity. Searches were conducted across multiple databases and platforms using Boolean operators and appropriate filters where available.

1. **Pubmed**

Search String:
(Parkinson's[MeSH]) AND (("drug therapy") OR (treatment) OR (medication) OR (antiparkinson*) OR ("dopamine replacement therapy") OR (dopamin*) OR (levodopa) OR ("dopamine agonist*") OR ("dopamine agent*") OR ("non pharmacological treatment") OR ("deep brain stimulation") OR ("dopamine antagonist*")) AND (("financial abilit*") OR ("financial capacit*") OR ("financial decision making") OR ("financial decision-making") OR ("financial capability") OR ("financial manag*"))
Filters: English language
Results: 10

2. **Web Of Science**

Search String:
ALL=(Parkinson's Disease) AND ALL=("drug therapy" OR treatment OR medication OR antiparkinson* OR "dopamine replacement therapy" OR dopamin* OR levodopa OR "dopamine agonist*" OR "dopamine agent*" OR "dopamine antagonist*" OR "deep brain stimulation" OR "cognitive therapy" OR "non pharmacological tratment*") AND ALL=("financial abilit*" OR "financial capacit*" OR "financial decision making" OR "financial decision-making" OR "financial capabilit*" OR "financial manag*")
Filter: None
Results: 6

3. **Cochrane Library**

Search String:
("Parkinsons disease") AND ("dopamine agonist" OR levodopa "drug therapy" OR treatment OR medication OR antiparkinson OR "dopamine replacement therapy" OR "dopamine agent" OR "dopamine antagonist" OR "deep brain stimulation" OR "cognitive therapy" OR "non pharmacological" tratment) AND ("financial ability" OR "financial capacity" OR "financial decision making" OR "financial decision-making" OR "financial capability" OR "financial management")
Search Fields: Title, Abstract, Keywords
Results: 3

4. **ProQuest**

Search String:
(("Parkinson's Disease" OR Parkinson OR PD) AND (medication OR treatment OR antiparkinson* OR "dopamine replacement therapy" OR dopamin* OR levodopa OR "dopamine agonist*" OR "dopamine

agent*" OR "dopamine antagonist*" OR "Deep Brain Stimulation" OR "Cognitive Therapy" OR "Non-pharmacological Treatment") AND ("financial abilit*" OR "financial capacit*" OR "financial decision making" OR "financial decision-making" OR "financial capabilit*" OR "financial management"))
Filters: Peer-reviewed, Scholarly Journals, English
Results: 1.657

5. **eBook Open Access (OA) Collection (EBSCOhost)**

Search String:
(parkinson's disease or parkinson disease or parkinsons disease or pd or parkinsons or parkinsonism) AND (therapy OR treatment OR medication OR antiparkinson* OR "dopamine replacement therapy" OR dopamin* OR levodopa OR "dopamine agonist*" OR "dopamine agent*" OR "dopamine antagonist*" OR "deep brain stimulation" OR "cognitive therapy" OR "non pharmacological") AND ("financial abilit*" OR "financial capacit*" OR "financial decision making" OR "financial decision-making" OR "financial capabilit*" OR "financial manag*")
Results: 0

6. **Scopus**

Search String:
(("Parkinson's Disease" OR parkinson OR pd) AND (medication OR treatment OR antiparkinson* OR "dopamine replacement therapy" OR dopamin* OR levodopa OR "dopamine agonist*" OR "dopamine agent*" OR "dopamine antagonist*" OR "Deep Brain Stimulation" OR "Cognitive Therapy" OR "Non-pharmacological Treatment" OR "Non pharmacological Treatment") AND ("financial abilit*" OR "financial capacit*" OR "financial decision making" OR "financial decision-making" OR "financial capabilit*" OR "financial management"))
Filters: English; Source type: Journal
Results: 1004

7. **ScienceDirect (Elsevier)**

Search String:
(("Parkinson's Disease") AND (treatment OR antiparkinsonian OR "dopamine replacement therapy" OR "Deep Brain Stimulation" OR "Non pharmacological Treatment") AND ("financial ability" OR "financial capacity" OR "financial decision-making")
Notes: Search adapted to conform to maximum 8 Boolean terms
Filter: Subscribed journals
Results: 33

8. **ERIC.**

Search String:
("Parkinson's Disease" OR Parkinson's) AND (drug therapy OR treatment OR "cognitive therapy" OR "non-pharmchological" OR "deep brain stimulation" OR medication OR antiparkinson* OR dopamine replacement therapy OR dopamin* OR levodopa OR dopamine agonist* OR dopamine agent* OR

dopamine antagonist*) AND (financial abilit* OR financial capacit* OR financial decision making OR financial decision-making OR financial capability OR financial manag*)
Filters: Peer-reviewed
Results: 11

9. **GoogleScholar**

Search String:
(("Parkinson's Disease" OR parkinson OR pd) AND (medication OR treatment OR antiparkinson* OR "dopamine replacement therapy" OR dopamin* OR levodopa OR "dopamine agonist*" OR "dopamine agent*" OR "dopamine antagonist*" OR "Deep Brain Stimulation" OR "Cognitive Therapy" OR "Non-pharmacological Treatment" OR "Non pharmacological Treatment") AND ("financial abilit*" OR "financial capacit*" OR "financial decision making" OR "financial decision-making" OR "financial capabilit*" OR "financial management"))
Results: 102
